# Sex differences in obstructive sleep apnea: a population-based study from northeastern Germany

**DOI:** 10.1007/s11325-026-03632-z

**Published:** 2026-02-28

**Authors:** Kora Steinmetz, Markus Krüger, Anne Obst, Ralf Ewert, Thomas Penzel, Ingo Fietze, Bernd Kordaß, Till Ittermann, Chia-Jung Busch, Christian Scharf, Sylvia Stracke, Beate Stubbe, Amro Daboul

**Affiliations:** 1 Department of Prosthodontics, Gerodontology and Biomaterials, University Medicine Greifswald, 17489 Greifswald, Germany; 2 Department for Internal Medicine B, University Medicine Greifswald, 17489 Greifswald, Germany; 3 Sleep Center, University Hospital Charité Berlin, 10117 Berlin, Germany; 4 Institute for Community Medicine, University Medicine Greifswald, 17475 Greifswald, Germany; 5 Department of Otorhinolaryngology, Head and Neck Surgery, University Medicine Greifswald, 17489 Greifswald, Germany; 6 Department of Internal Medicine A, University Medicine Greifswald, 17489 Greifswald, Germany

**Keywords:** Obstructive sleep apnea, Sex differences, Polysomnography, REM sleep, Epworth sleepiness scale

## Abstract

**Purpose:**

Obstructive sleep apnea (OSA) is a common sleep-related breathing disorder that is diagnosed more frequently in men. The aim of this population-based cross-sectional study was to describe sex-specific differences in sleep stage distribution and subjective daytime sleepiness in individuals with OSA. Special attention was given to the distribution of the apnea-hypopnea index (AHI) in rapid eye movement (REM) and non-REM sleep.

**Methods:**

This study analyzed data from the SHIP-TREND-0 cohort, a population-based study from northeastern Germany. Standardized polysomnography identified 604 participants with an AHI ≥ 5 (395 men, 209 women) who were included in this study. In addition, daytime sleepiness was recorded using the Epworth Sleepiness Scale (ESS). Sex differences were analyzed descriptively and evaluated according to effect sizes.

**Results:**

Compared to males, females were on average older and had higher BMI values. Women showed descriptively higher AHI values in REM sleep than in non-REM sleep, whereas men exhibited a more balanced distribution of AHI across sleep stages. Women had a longer REM sleep latency (*d* = 0.36).

**Conclusions:**

Our results indicate sex-specific differences in the distribution of obstructive events across sleep stages. The AHI during REM sleep should receive greater consideration in the diagnosis and assessment of severity, particularly in women. Further population-based studies are needed to confirm these findings and evaluate their clinical relevance.

**Supplementary Information:**

The online version contains supplementary material available at 10.1007/s11325-026-03632-z.

## Introduction

Obstructive sleep apnea (OSA) is a common sleep-related breathing disorder characterized by recurrent episodes of partial or complete upper airway obstruction during sleep, leading to intermittent hypoxemia and sleep fragmentation [1, 2]. A systematic review reported a prevalence of 9% to 38% in the adult general population, based primarily on studies from Europe and North America [3]. OSA is associated with increased morbidity from cardiovascular disease, metabolic dysfunction, and all-cause mortality [4, 5]. In addition, an increased risk of traffic accidents has been demonstrated [6].

The most important risk factors for OSA are male sex, older age, obesity, smoking and alcohol consumption [7]. Overall, men have a fat distribution pattern associated with greater risk, which at least partially explains the higher prevalence in this group [8, 9]. In women, the hormonal situation also plays an important role. Premenopausal women are less frequently affected, whereas the risk increases after menopause [10–13]. Various factors may contribute to the increased prevalence of sleep disturbances and OSA in postmenopausal women, including hormonal changes, age-related alterations in sleep architecture, comorbid conditions such as depression, and vasomotor symptoms like hot flashes and night sweats. These factors can lead to sleep fragmentation and reduced sleep quality, which may explain the increased prevalence of OSA in this population [14]. Other sources emphasize possible direct hormonal effects on the ventilatory control system, as well as hormonally mediated changes in fat distribution [12, 13]. However, the underlying mechanisms of the increased OSA risk after menopause are not fully understood.

Men are at higher risk of developing OSA, with population-based studies estimating the male-to-female ratio of approximately 1.5:1 [15]. However, women have historically been underrepresented in OSA research, as many earlier studies included only male participants [16]. One of the first major population-based studies that systematically included women was not published until 1993 as part of the Wisconsin Sleep Cohort Study [17]. Until then, most available data came from clinical samples, resulting in selection bias and an underestimation of the true prevalence of OSA in women [18, 19].

In addition to these factors, symptom perception often differs between women and men. While snoring and witnessed apneas, typically reported by bed partners, are more commonly attributed to men, women more frequently describe symptoms such as fatigue, depression and anxiety [16, 20]. This may contribute to later diagnosis and underdiagnosis in women with OSA [21, 22].

One phenomenon that has attracted growing research interest is the sex-specific distribution of obstructive events across different sleep stages. While men typically show a relatively stable AHI throughout the night, women are more likely to exhibit REM-predominant OSA, with apneas and hypopneas occurring more frequently during REM sleep [23, 24]. The clinical relevance of this pattern is a subject of ongoing discussion, as a high REM-AHI may be linked to elevated cardiovascular risk [25–27].

Much of the existing literature relies on clinical cohorts of previously diagnosed patients. This carries the risk of selection bias, as women with atypical symptoms in particular are less likely to receive a diagnosis and are therefore underrepresented in these samples [28, 29].

The aim of this study is to analyze and report sex-specific differences in patients with OSA. To investigate this hypothesis, we utilized data from the Study of Health in Pomerania (SHIP), a large-scale, longitudinal, population-based health study conducted in northeastern Germany. SHIP was designed to examine the prevalence of chronic diseases, associated risk factors, and their interactions within the general adult population. Data collection involves standardized face-to-face interviews, self-administered questionnaires, physical examinations, and the acquisition of various biospecimens. The study encompasses the SHIP-TREND cohort - an independent cohort established in 2008 (TREND-0: 2008–2012). Participants were selected through random sampling from regional population registries and underwent comprehensive, standardized clinical assessments, structured interviews, and administration of validated questionnaires [30]. This study takes an explorative, descriptive approach to examine sex-specific differences in OSA. The focus lies on sleep stages, the distribution of obstructive events across sleep stages, and the subjective perception of symptoms as measured by the Epworth Sleepiness Scale (ESS). The combination of objective polysomnographic parameters and subjective information on daytime sleepiness should help to identify sex-related differences that could contribute to a delayed or more difficult diagnosis.

## Participants and methods

### Data basis

This cross-sectional study is based on data from SHIP, a population-based cohort study conducted in northeastern Germany. SHIP recruited a large number of participants to collect comprehensive information on various health-related parameters. For the present analysis, data from the baseline examination of the SHIP-TREND cohort, known as SHIP-TREND-0, were used exclusively. This was the first SHIP cohort to include sleep-related data, obtained through standardized questionnaires and overnight polysomnography. Data collection for SHIP-TREND-0 took place between 2008 and 2012. For the SHIP-TREND-0 cohort, a random sample of 10,000 individuals aged 22 to 81 years was drawn from the general population, stratified by sex and age. A detailed overview of the data collected in SHIP-TREND is provided by Völzke et al. [30].

### Participants

A total of 4,420 individuals consented to participate in SHIP-TREND-0. Of these, 1,264 underwent standardized overnight polysomnography at the local sleep laboratory. Complete datasets were available for 1,209 participants. Of these, 650 were male and 559 were female. Overall, 604 individuals (50.0%) had an apnea-hypopnea index (AHI, events per hour) ≥ 5. The prevalence of AHI ≥ 5 affected 60.8% of men and 37.4% of women.

For the present analysis, only participants with an apnea-hypopnea index ≥ 5 were included, resulting in a total sample of 604 individuals. Of these, 395 (65%) were male and 209 (35%) female. The AHI quantifies the number of apneas and hypopneas per hour of sleep and is commonly used to classify the severity of OSA. An AHI of ≥ 5 was defined as mild OSA, ≥ 15 events per hour as moderate OSA, and values ≥ 30 as severe OSA. REM-related parameters were excluded for 17 participants because they had no measurable REM sleep. A total of 577 participants completed the ESS questionnaire.

### Polysomnography & data collection

Participants attended a single-night, laboratory-based polysomnography (PSG) (Alice 5 System; Philips Respironics, Eindhoven, The Netherlands) in Greifswald, Germany. Sensor placement was performed according to the 2007 American Academy of Sleep Medicine (AASM) rules [31].

Recordings included six electroencephalogram (EEG) channels, two electrooculogram (EOG) channels, two electromyogram (EMG) channels positioned at the chin and tibialis muscles, one electrocardiogram (ECG) channel, respiratory inductive plethysmography, a nasal pressure sensor, pulse oximetry and a body position sensor (further details on the sleep examinations within SHIP can be found elsewhere; Fietze et al. [32], Stubbe et al. [33]). An apnea was defined as a reduction in airflow of at least 90% lasting at least 10 s, with this reduction present for at least 90% of the event duration. A hypopnea was defined as either a reduction in airflow of at least 30% for at least 10 s associated with an oxygen desaturation of at least 4%, or a reduction in airflow of at least 50% associated with an oxygen desaturation of at least 3% or an arousal [31, 32].

### Analyzed variables

Sex-specific differences were examined based on selected demographic and polysomnographic parameters. Age was treated as a categorical variable and divided into three groups: ≤ 39 years, 40–59 years, and ≥ 60 years. Additional parameters included sex (male/female) and body mass index (BMI). The BMI classification followed the German guideline on sleep-related breathing disorders and was divided into the following categories: 18.5–24.99 kg/m² (normal weight), 25.0–29.99 kg/m² (pre-obesity), 30.0–34.99 kg/m² (obesity grade I), 35.0–39.99 kg/m² (obesity grade II), and ≥ 40 kg/m² (obesity grade III) [34]. No participants with underweight (< 18.5 kg/m²) were present in the sample.

Total Sleep Time (TST) refers to the overall sleep duration in minutes and comprises the time spent in REM and non-REM sleep. Wake After Sleep Onset (WASO) is defined as the number of minutes participants remained awake after initially falling asleep. Sleep latency describes the time from lights out to sleep onset, while REM latency refers to the duration between sleep onset and the first occurrence of REM sleep. Sleep efficiency is calculated as the ratio of total sleep time to time in bed, expressed as a percentage. REM-predominant OSA was defined as a higher AHI during REM sleep than during non-REM sleep (AHI-REM > AHI-nREM).

The categorization of age groups as well as the selection and presentation of polysomnographic parameters followed the methodology described by Vagiakis et al. [35]. This allows for a structured comparison with the sex-specific findings reported in that study and reflects an approach commonly used in the literature.

As an additional parameter, the ESS, a standardized questionnaire for assessing daytime sleepiness, was used [36]. An ESS score greater than 10 was considered indicative of excessive daytime sleepiness [37]. 

### Statistical analysis

To further explore sex-specific differences, a range of polysomnographic parameters was included. These were analyzed descriptively and assessed using Cohen’s d as a measure of effect size. Cohen’s d expresses the difference between two means in terms of standard deviation units [38]. Given the exploratory nature of this study, results are primarily interpreted based on effect sizes rather than formal hypothesis testing.

Statistical analyses were performed using R (version 4.4.2) [39] in RStudio (version 24.09.1 Build 394) [40] and the packages haven [41] and dplyr [42].

## Results

### Age, sex, and BMI distribution

The sample consisted of 395 men and 209 women, corresponding to a male-to-female ratio of approximately 1.9:1. Women had a mean age of 61 years (SD = 9), whereas men had a mean age of 56 years (SD = 12). Mean BMI was higher in women (31.1 kg/m^2^, SD = 5.6) than in men (29.8 kg/m^2^, SD = 4.5). Table 1 presents the age distribution of participants across age categories. The largest proportion of male participants (50.4%) was in the 40–59-year age group, whereas the majority of female participants (58.4%) were aged 60 years or older. The smallest proportion of participants in both sexes was found in the ≤ 39-year age group, accounting for 8.6% of men and only 0.5% of women.

**Table 1:** Age distribution by sex (*N* = 604)

Age Groups	Male (*n* = 395)	Female (*n* = 209)
39 years or younger	34 (8.6%)	1 (0.5%)
40–59 years	199 (50.4%)	86 (41.1%)
60 years or older	162 (41.0%)	122 (58.4%)

Data are number (percentages). *n* = number of participants

As shown in Table 2, fewer than 12% of participants in both sexes were of normal weight (BMI 18.5–24.99 kg/m^2^). The largest proportion of men (44.8%) were in the pre-obesity category (BMI 25–29.99 kg/m²), while the largest proportion of women(35.9%) were in obesity grade I. Overall, women were more likely than men to have a BMI in the obese range.

**Table 2:** BMI categories by sex (*N* = 604)

BMI (kg/m^2^)	Male (*n* = 395)	Female (*n* = 209)
18.5–24.99	44 (11.1%)	23 (11.0%)
25–29.99	177 (44.8%)	68 (32.5%)
30–34.99	129 (32.7%)	75 (35.9%)
35–39.99	33 (8.4%)	27 (12.9%)
≥ 40	12 (3.0%)	16 (7.7%)

Data are number (percentages). *BMI* body mass index, *n* number of participants

### Sex differences in sleep parameters

REM latency was longer in women, with a mean of 167 min compared to 137 min in men (*Cohen’s d* = 0.36). REM sleep duration was longer in male participants, averaging 50 min compared to 43 min in female participants (*d* = 0.27). Sleep latency and sleep efficiency showed comparable mean values between sexes, with effect sizes of *d* = 0.20 for both parameters. For TST, non-REM sleep, and WASO, effect sizes were below *d* = 0.20 (Table 3).

**Table 3:** Comparison of polysomnographic sleep parameters by sex

Parameter	Male (*n* = 395)	Female (*n* = 209)	Mean Difference	Cohen’s d
TST in min	369 (68) [362; 375] (73–525)	362 (64) [354; 371] (167–492)	7	0.10
REM sleep in min	50 (27) [48; 53] (0–140)	43 (25) [40; 47] (0–130)	7	0.27
nREM sleep in min	318 (58) [313; 324] (72–462)	319 (58) [311; 326] (167–443)	−0.3	0.004
WASO in min	66 (47) [61; 70] (2–303)	73 (48) [67; 80] (1–236)	−7	0.15
Sleep latency in min	14 (17) [13; 16] (0–160)	17 (15) [15; 20] (0–87)	−3	0.20
^a^ Rem latency in min	137 (79) [129–145] (0–459)	167 (94) [154; 180] (2–456)	−30	0.36
Sleep efficiency in %	81 (13) [79; 82] (15–99)	78 (12) [76; 80] (43–98)	3	0.20

Data are mean (SD) [CI] (range). *TST* total sleep time, *REM* rapid eye movement, *nREM* non-rapid eye movement, *WASO* wake after sleep onset, *SD* standard deviation, *CI* confidence interval, *n* number of participants. ^a^ Male *n* = 383, Female *n* = 204

### AHI analyses

Women showed higher mean AHI values during REM sleep, whereas men had higher values during non-REM sleep. Overall, effect sizes for sex differences in AHI were small (*Cohen’s d* = 0.25–0.34) (Table 4).

**Table 4:** Mean AHI values by sleep stage and sex

Parameter	Male (*n* = 395)	Female (*n* = 209)	Mean Difference	Cohen’s d
AHI	21 (17) [19; 23] (5–92)	17 (14) [15; 19] (5–82)	4	0.25
^a^ AHI during REM	20 (19) [18; 22] (0–105)	27 (20) [24; 29] (0–86)	−7	0.34
AHI during nREM	21 (17) [19; 23] (1–93)	16 (14) [14; 18] (1–82)	5	0.32

Data are mean (SD) [CI] (range). *AHI* apnea-hypopnea index, *REM* rapid eye movement, *nREM* non-rapid eye movement, *SD* standard deviation, *CI* confidence interval, *n* number of participants. ^a^ Male *n* = 383, Female *n* = 204

These stage-specific differences were also reflected in the distribution of individual AHI patterns (Table 5). In 68.1% of women, the AHI was higher during REM sleep than during non-REM sleep, whereas this pattern was observed in only 48.3% of men. Conversely, a higher AHI in non-REM sleep was observed in 51.7% of men and 31.9% of women.

**Table 5:** Distribution of higher AHI in REM or non-REM sleep by sex (*N* = 587)

Distribution	Male (*n* = 383)	Female (*n* = 204)
AHI-REM > AHI-nREM	185 (48.3%)	139 (68.1%)
AHI-REM < AHI-nREM	198 (51.7%)	65 (31.9%)

Data are number (percentages). *AHI* apnea-hypopnea index, *REM* rapid eye movement, *nREM* non-rapid eye movement, *n* number of participants

### ESS scores across AHI categories

As shown in Table 6, daytime sleepiness was consistently higher in men than in women, regardless of OSA severity. In all three categories, the average ESS score in men was at least one point higher than in women (*Cohen’s d* ≥ 0.30). These differences were below the minimal clinically important difference for the ESS, which has been reported to be approximately 2 to 3 points [43]. Although some participants reached ESS scores above 10, mean values in all groups remained below this threshold.

**Table 6:** Epworth Sleepiness Scale scores by sex and OSA severity (*n* = 577)

AHI	ESS		Mean Difference	Cohen’s d
	Male	Female		
total	7.4 (3.3) [7.0; 7.7] (0–19) *n* = 381	6.3 (3.3) [5.8; 6.7] (0–18) *n* = 196	1.0	0.33
5 ≤ AHI < 15	7.2 (3.4) [6.7; 7.7] (0–17) *n* = 185	6.2 (3.1) [5.7; 6.8] (0–17) *n* = 118	1.0	0.30
15 ≤ AHI < 30	7.4 (3.3) [6.8; 8.0] (1–19) *n* = 117	6.3 (3.4) [5.4; 7.2] (0–18) *n* = 55	1.1	0.33
30 ≥ AHI	7.6 (2.9) [6.9; 8.2] (2–17) *n* = 79	6.6 (3.7) [5.1; 8.1] (1–14) *n* = 23	1.0	0.31

Data are mean (SD) [CI] (range). *AHI* apnea-hypopnea index, *ESS* Epworth Sleepiness Scale, *SD* standard deviation, *CI* confidence interval, *n* number of participants

## Discussion

Our cohort, drawn from the Study of Health in Pomerania (SHIP), represents a population-based sample from northeastern Germany. This design reduces referral bias typical of clinical cohorts. This study indicated several differences in OSA characteristics and sleep parameters between men and women. Women more frequently exhibited REM-predominant OSA, with a higher AHI during REM sleep. Although men showed higher overall AHI descriptively, women had a higher mean AHI during REM than the men. This was reversed in the non-REM sleep. Additionally, women demonstrated longer REM latency and shorter REM duration. Other sleep parameters, including total sleep time, sleep efficiency, and WASO showed minimal sex differences.

Women in our cohort tended to be older and more frequently obese than men, yet showed a lower overall AHI. This indicates a higher OSA burden in men, despite the presence of more obesity-related risk factors in women.

The higher prevalence of REM-predominant OSA in women observed in our cohort is consistent with previous studies. Bahammam et al. [23] reported that REM-predominant OSA was more common in women and suggested hormonal and age-related influences. Similarly, Mehra et al. [44] noted sex differences in REM-related respiratory disturbances, with female patients exhibiting higher REM-AHI values than males. These findings have been replicated in several other studies [26].

Our observation that men had higher overall AHI aligns with the well-established finding that OSA tends to present with greater severity in men [7]. The commonly reported 1.5:1 male-to-female ratio likely reflects both physiological differences and diagnostic bias [15]. Basoglu & Tasbakan [45] suggest that men are more likely to meet conventional AHI-based thresholds, whereas women often present with REM-dominant patterns. This may contribute to women being less likely to be captured by these criteria.

The longer REM latency and shorter REM duration in women observed in our study are consistent with findings from Hirotsu et al. [46], who also reported altered REM architecture in female patients, although the shorter REM duration in their study was not statistically significant. In our cohort, ESS scores were slightly higher in men, but the observed differences remained below the established threshold for clinical relevance. Previous reports have indicated that women with OSA often report less daytime sleepiness than men, even in the presence of moderate or severe OSA. Sex differences in subjective sleepiness have been proposed as a factor contributing to the underdiagnosis of OSA in women [47].

Beyond subjective symptoms, sex-related differences were also observed in general sleep parameters. In our cohort, women exhibited slightly lower sleep efficiency and longer WASO. Previous studies have reported similar findings, suggesting that women may experience lighter or more fragmented sleep [14]. These differences may contribute to the divergence between AHI and symptom reporting in women.

Despite being older and more frequently obese, women in our cohort had lower overall AHI than men. This discrepancy suggests that AHI alone may be an insufficient marker of OSA severity in female patients.

Comparisons with other population-based studies highlight meaningful differences. The finding that women are older and have lower overall AHI is consistent across various studies, whereas results regarding BMI are less clear. Several cohorts, including those by Vagiakis et al. [35] and Votteler et al. [48], reported women with BMI similar to men. In contrast, our cohort observed that women tended to have higher BMI than men, which is consistent with findings from a clinical study in Japanese patients by Yukawa et al. [49].

These variations suggest that sex-specific OSA patterns may vary by region or methodology. This aligns with Ye et al. [22], who reviewed data from multiple countries and concluded that standard diagnostic thresholds may not adequately capture female OSA phenotypes, potentially contributing to underrecognition in women.

A key strength of this study is its use of a population-based sample, which limits referral and selection bias. Standardized data collection and scoring procedures improve internal consistency, and the relatively large sample size supports generalizability within the region.

However, several limitations should be noted. First, our analysis is explorative in nature and was based on descriptive statistics without formal hypothesis testing, limiting the interpretability of observed differences. Second, the sample was drawn from a specific geographic and ethnic population, which may affect generalizability. Third, our use of a fixed AHI threshold (≥ 5) does not account for clinical subtypes, such as REM-related or positional OSA. Furthermore, the lack of a universally accepted definition for REM-predominant OSA may limit the comparability of our findings with other studies. Finally, self-reported measures like the ESS have known limitations and may not capture daytime impairment or reflect sex-specific symptom patterns.

Sex-specific differences might be relevant for diagnostic strategies and CPAP titration procedures performed outside the sleep laboratory. As highlighted by Martínez-García and Labarca [19], current diagnostic pathways and treatment algorithms are largely based on studies in middle-aged men and may not adequately capture female OSA phenotypes. In addition, current guidelines lack sex-specific orientation. The current German guideline on sleep-related breathing disorders in adults does not include sex-specific diagnostic or therapeutic recommendations [50]. Given that more than 200 million women worldwide are estimated to have mild OSA [51], greater attention to sex-specific aspects in diagnostic approaches and CPAP titration protocols could help to ensure better recognition and treatment in women.

### Conclusion

In conclusion, women in our population-based sample more frequently exhibited REM-predominant OSA, with higher AHI values during REM sleep despite lower overall AHI. These findings suggest that current diagnostic approaches, which rely heavily on overall AHI and classic symptoms, may not fully characterize OSA in women.

Overall, our results provide population-based, stage-specific polysomnographic data showing that sex differences in OSA are not limited to overall AHI but also involve distinct patterns in the distribution of obstructive events and REM-related sleep characteristics. Taken together, these findings support the need for a more nuanced and sex-specific approach to OSA assessment that considers REM-related patterns to ensure accurate diagnosis and treatment.

## Supplementary Information

Below is the link to the electronic supplementary material.

Supplementary Material 1 (DOCX 32.0 KB)

## Data Availability

Due to the informed consent provided by study participants, SHIP data cannot be made publicly available. Nonetheless, researchers who fulfill the necessary criteria for accessing confidential data may apply for data access via the SHIP transfer portal: https://transfer.ship-med.uni-greifswald.de/FAIRequest/.

## Declarations

### Generative AI and AI-assisted technologies in the writing process

During the preparation of this work, the authors used ChatGPT (OpenAI) in order to improve the readability and language of the manuscript. After using this tool, the authors reviewed and edited the content as needed and take full responsibility for the content of the published article.
