# Supplementary material for: Sex differences in obstructive sleep apnea: a population-based study from northeastern Germany
Source: Sleep Breath. 2026 Feb 28;30(1):70. doi: 10.1007/s11325-026-03632-z (PMC12950006; doi:10.1007/s11325-026-03632-z)
Supplement: Supplementary file 1 — Supplementary Material 1 (DOCX 32.0 KB) [file 11325_2026_3632_MOESM1_ESM.docx]

**Table S1.** Age distribution of the total PSG sample by sex (N = 1,209)

| Age Groups | Male (n = 650) | Female (n = 559) |
| --- | --- | --- |
| 39 years or younger | 128 (19.7 %) | 88 (15.7 %) |
| 40-59 years | 307 (47.2 %) | 273 (48.8 %) |
| 60 years or older | 215 (33.1 %) | 198 (35.4 %) |

Note: Data are number (percentages). n = number of participants

**Table S2.** BMI categories of the total PSG sample by sex (N = 1,208 ^a^)

| BMI (kg/m^2^) | Male (n = 650) | Female (n = 558) |
| --- | --- | --- |
| <18.5 | 0 (0.0 %) | 2 (0.4 %) |
| 18.5 – 24.99 | 123 (18.9 %) | 166 (29.7 %) |
| 25 – 29.99 | 303 (46.6 %) | 193 (34.6 %) |
| 30 – 34.99 | 174 (26.8 %) | 135 (24.2 %) |
| 35 – 39.99 | 36 (5.5 %) | 40 (7.2 %) |
| ≥ 40 | 14 (2.2 %) | 22 (3.9 %) |

Note: Data are number (percentages). BMI = body mass index; n = number of participants.

^a^ BMI data were missing for one female participant.

**Table S3.** Comparison of polysomnographic sleep parameters by sex: unadjusted t-tests and effect sizes

| Parameter | Male  (n = 395) | Female  (n = 209) | Mean Difference | p (t-test) | Cohen’s *d* |
| --- | --- | --- | --- | --- | --- |
| TST in min | 369 (68) | 362 (64) | 7 | 0.231 | 0.10 |
| REM sleep in min | 50 (27) | 43 (25) | 7 | 0.001 | 0.27 |
| nREM sleep in min | 318 (58) | 319 (58) | -0.3 | 0.958 | 0.004 |
| WASO in min | 66 (47) | 73 (48) | -7 | 0.074 | 0.15 |
| Sleep latency in min | 14 (17) | 17 (15) | -3 | 0.017 | 0.20 |
| ^a^ Rem latency in min | 137 (79) | 167 (94) | -30 | < 0.001 | 0.36 |
| Sleep efficiency in % | 81 (13) | 78 (12) | 3 | 0.020 | 0.20 |

Note: Data are mean (SD). TST = total sleep time; REM = rapid eye movement; nREM = non-rapid eye movement; WASO = wake after sleep onset; n = number of participants. ^a^Male n=383, Female n=204.

**Table S4.** Comparison of polysomnographic sleep parameters by sex: linear regression analyses adjusted for age and BMI

| Parameter | β (sex) | R²_p_ | p (adjusted model) |
| --- | --- | --- | --- |
| TST in min | 2.0 | 0.06 | 0.719 |
| REM sleep in min | -3.7 | 0.05 | 0.099 |
| nREM sleep in min | 5.7 | 0.03 | 0.256 |
| WASO in min | -0.1 | 0.11 | 0.978 |
| Sleep latency in min | 2.0 | 0.02 | 0.164 |
| ^a^ Rem latency in min | 27.8 | < 0.01 | < 0.001 |
| Sleep efficiency in % | -0.4 | 0.12 | 0.684 |

Note: TST = total sleep time; REM = rapid eye movement; nREM = non-rapid eye movement; WASO = wake after sleep onset. ^a^ Male n=383, Female n=204.

**Table S5.** Mean AHI values by sleep stage and sex: unadjusted t-tests and effect sizes

| Parameter | Male  (n = 395) | Female  (n = 209) | Mean Difference | p (t-test) | Cohen’s *d* |
| --- | --- | --- | --- | --- | --- |
| AHI | 21 (17) | 17 (14) | 4 | 0.002 | 0.25 |
| ^a^ AHI during REM | 20 (19) | 27 (20) | -7 | < 0.001 | 0.34 |
| AHI during nREM | 21 (17) | 16 (14) | 5 | < 0.001 | 0.32 |

Note: Data are mean (SD). AHI = apnea-hypopnea index; REM = rapid eye movement; nREM = non-rapid eye movement; n = number of participants. ^a^ Male n=383, Female n=204.

**Table S6.** Mean AHI values by sleep stage and sex: linear regression analyses adjusted for age and BMI

| Parameter | β (sex) | R²_p_ | p (adjusted model) |
| --- | --- | --- | --- |
| AHI | -6.3 | 0.03 | < 0.001 |
| ^a^ AHI during REM | 4.2 | 0.01 | 0.012 |
| AHI during nREM | -7.6 | 0.03 | < 0.001 |

Note: AHI = apnea-hypopnea index; REM = rapid eye movement; nREM = non-rapid eye movement. ^a^ Male n=383, Female n=204.

**Table S7.** Epworth Sleepiness Scale scores by sex and OSA severity: unadjusted t-tests and effect sizes (*n* = 577)

| Parameter | ESS | | Mean Difference | p (t-test) | Cohen’s *d* |
| --- | --- | --- | --- | --- | --- |
|  | Male | Female |  |  |  |
| total | 7.4 (3.3) n = 381 | 6.3 (3.3) n = 196 | 1.0 | < 0.001 | 0.33 |
| 5 ≤ AHI <15 | 7.2 (3.4) n = 185 | 6.2 (3.1) n = 118 | 1.0 | 0.009 | 0.30 |
| 15≤ AHI < 30 | 7.4 (3.3) n = 117 | 6.3 (3.4) n = 55 | 1.1 | 0.048 | 0.33 |
| 30 ≥ AHI | 7.6 (2.9) n = 79 | 6.6 (3.7) n = 23 | 1.0 | 0.257 | 0.31 |

Note: Data are mean (SD). AHI = apnea-hypopnea index; ESS = Epworth Sleepiness Scale; n = number of participants

**Table S8.** Epworth Sleepiness Scale scores by sex and OSA severity: linear regression analyses adjusted for age and BMI (*n* = 577)

| Parameter | β (sex) | R²_p_ | p (adjusted model) |
| --- | --- | --- | --- |
|  |  |  |  |
| total | -1.1 | 0.01 | < 0.001 |
| 5 ≤ AHI <15 | -0.9 | 0.01 | 0.026 |
| 15≤ AHI < 30 | -1.4 | < 0.01 | 0.016 |
| 30 ≥ AHI | -0.8 | 0.02 | 0.270 |

Note: AHI = apnea-hypopnea index; ESS = Epworth Sleepiness Scale; n = number of participants
